# The role of ultrasound-defined tenosynovitis and synovitis in the prediction of rheumatoid arthritis development

**DOI:** 10.1093/rheumatology/key025

**Published:** 2018-04-03

**Authors:** Ilfita Sahbudin, Luke Pickup, Peter Nightingale, Gina Allen, Zaeem Cader, Ruchir Singh, Paola de Pablo, Christopher D Buckley, Karim Raza, Andrew Filer

**Affiliations:** 1 Institute of Inflammation and Ageing, University of Birmingham, Birmingham, UK; 2 University Hospitals Birmingham NHS Foundation Trust, Birmingham, UK; 3 Sandwell and West Birmingham Hospitals NHS Trust, Birmingham, UK; 4 Rheumatology Department, Wolfson Computer Laboratory, University Hospital Birmingham NHS Foundation Trust, Birmingham, UK; 5 Green Templeton College, University of Oxford, Oxford, UK; 6 Division of Gastroenterology and Hepatology, Addenbrooke’s Hospital, University of Cambridge, Cambridge, UK; 7 MRC-ARUK Centre for Musculoskeletal Ageing Research, University of Birmingham, Birmingham, UK

**Keywords:** tenosynovitis, ultrasound, rheumatoid arthritis, synovitis

## Abstract

**Objectives:**

Tenosynovitis (TS) is common in early arthritis. However, the value of US-defined TS in predicting RA development is unclear. We assessed the predictive utility of US-defined TS alongside US-defined synovitis and clinical and serological variables in a prospective cohort of early arthritis patients.

**Methods:**

One hundred and seven patients with clinically apparent synovitis of one or more joint and symptom duration ⩽3 months underwent baseline clinical, laboratory and US assessment of 19 bilateral joint sites and 16 bilateral tendon compartments. Diagnostic outcome was determined after 18 months, applying the 2010 ACR/EULAR classification criteria for RA. The predictive values of US-defined TS for persistent RA were compared with those of US-defined synovitis, clinical and serological variables.

**Results:**

A total of 4066 US joint sites and 3424 US tendon compartments were included in the analysis. Forty-six patients developed persistent RA, 17 patients developed non-RA persistent disease and 44 patients had resolving disease at follow-up. US-defined TS in at least one tendon compartment at baseline was common in all groups (RA 85%, non-RA persistent disease 71% and resolving 70%). On multi-variate analysis, US-defined digit flexor TS provided independent predictive data over and above the presence of ACPA and US-defined joint synovitis.

**Conclusion:**

US-defined digit flexor TS provided independent predictive data for persistent RA development in patients with early arthritis. The predictive utility of this tendon site should be further assessed in a larger cohort; investigators designing imaging-based predictive algorithms for RA development should include this tendon component as a candidate variable.

**Rheumatology key messages**

- US-defined digit flexor tenosynovitis provides independent predictive data for RA development in early arthritis patients.
- Clinicians should consider scanning digit flexor tendon sheaths alongside joints to enhance diagnostic confidence for RA in early arthritis clinics.

## Introduction

Initiation of immunosuppressant therapy during the early phases of RA alters the trajectory of disease progression [1]. However, distinguishing individuals who are at risk of progressing to RA from those whose disease will regress amongst patients presenting with clinical arthritis within 12 weeks of symptom onset remains a challenge.

Many current predictive algorithms for RA progression are based on clinical joint involvement, alongside clinical and serological variables [2]. Musculoskeletal US is a non-invasive and well-tolerated imaging technique and has been shown to improve the predictive ability of such algorithms [3, 4] due to the detection of subclinical synovitis [5].

Tenosynovitis (TS) is a well-recognized clinical feature of RA [6–9] and US is a reliable tool to assess TS in RA patients [10, 11]. However, the ability of US-defined TS to add data predictive of RA in patients with early clinically apparent synovitis is currently unknown.

In this study, we described the frequency and distribution of US-defined TS at multiple sites in patients with inflammatory arthritis of ⩽3 months symptom duration. We then determined whether US-defined TS provides predictive data over and above US-defined synovitis and other clinical and serological variables.

## Methods

### Patients and clinical assessments

Patients were recruited from the Birmingham Early Arthritis Clinic based in Rheumatology Departments at Sandwell and West Birmingham Hospitals National Health Service (NHS) Trust and University Hospitals Birmingham NHS Foundation Trust, UK. All patients were referred by their general practitioner to these two secondary care centres, which provide rheumatology service to a population of 1.3 million across Birmingham.

Consecutive DMARD-naïve patients with clinically detected synovitis of at least one joint and inflammatory joint symptom duration (pain and/or stiffness and/or swelling) of ⩽3 months were included. Patients who had joint symptoms attributed solely to degenerative joint disease were excluded. This study was conducted with the approval of the Solihull Local Research Ethics Committee and all patients gave written informed consent.

All consecutive patients who consented to this study were included in the analysis except for those who declined to continue follow-up before final diagnostic outcome data were available. One hundred and seven patients were reviewed at 1, 2, 3, 6, 12 and 18 months, and detailed clinical data were recorded at all visits, including DMARD treatments. Final diagnostic outcomes were determined at 18 month follow-up.

Patients were classified as having RA if they fulfilled cumulative 2010 ACR/EULAR [12] criteria by the 18-month visit. Patients were classified as having resolving disease at 18-month follow-up if they had no clinical evidence of joint synovial swelling, were not taking DMARDs and had not received steroid treatment (by any route) in the previous 3 months. Non-RA patients were classified according to established classification criteria, which were PsA, SLE and AS [13–15].

The following data were recorded at baseline: 68 tender and 66 swollen clinical counts, age, sex, symptom duration, early morning stiffness duration, medication, ESR, CRP, RF and ACPA status.

### Sonographic assessment

Within 24 h of clinical assessment, one experienced sonographer (A.F.) performed a blinded US assessment in a temperature-controlled radiology suite. Systematic multi-planar grey scale (GS) and power Doppler (PD) US examinations of 19 bilateral joint sites and 16 bilateral tendon compartments were performed based upon standard EULAR reference scans [11] using a Siemens Acuson Antares scanner (Siemens, Bracknell, UK) and multi-frequency (5–13 MHz) linear array transducers. The joint and tendon sites scanned are listed in supplementary Tables S1 and S2, available at *Rheumatology* online, respectively. For PD examinations, the pulse repetition frequency was adjusted to provide maximal sensitivity at the lowest possible value for each joint, resulting in pulse repetition frequencies of between 610 and 780. Examinations took between 50 and 60 min depending on disease extent and patient mobility.

US findings of GS synovitis and PD positivity were defined according to consensus definitions [16, 17]. GS synovitis and PD positivity in the MCP, PIP and MTP joints were graded from 0 to 3 as reported previously [3]. Synovitis in other joints was graded as: 0, normal; 1, mild; 2, moderate; and 3, severe, as previously reported [3].

GS and PD TS changes were defined and graded according to the OMERACT Ultrasound Task Force consensus definitions [11]. GS TS was defined as abnormal anechoic and/or hypoechoic (relative to tendon fibres) tendon sheath widening that was related to tenosynovial abnormal fluid and/or hypertrophy. PD TS was defined as the presence of peritendinous Doppler signal within the synovial sheath, seen in two perpendicular planes, excluding normal feeding vessels.

### Statistical analysis

#### Descriptive analysis

All data analyses were performed using IBM SPSS Statistics for Windows (Version 20.0.; IBM Corp., Armonk, NY, USA). Baseline clinical variables between groups were compared using Kruskal–Wallis or Fisher’s exact tests as appropriate. The proportions of patients with US-defined synovitis and TS between the outcome groups were compared using Fisher’s exact test. In descriptive analyses, *P* ⩽ 0.017 (0.05/3) was considered statistically significant after adjusting for the effect of multiple comparisons using the Bonferroni method.

#### Logistic regression and principal component analyses

The primary aim of this study was to identify the most parsimonious combination of US, clinical and serological variables that, when applied to a cohort of patients with early arthritis, identified patients progressing to RA by 18 months. All GS and PD US variables were binarized into absent (grade = 0) or present (grade ⩾1). Univariate logistic regression analysis was then performed to identify individual variables associated with the development of RA. Secondly, principal component analysis (PCA) was used to assess the degree of clustering amongst US joint and tendon variables and then clinical variables. The resulting selected variables were then used in a first multiple logistic regression analysis.

A second logistic regression model was then developed using systematic entry of US joint variable in order to confirm the independence of US-measured tendon and joint variables in prediction of RA. All independent clinical and serological variables were classified into categories as listed in supplementary Table S3, available at *Rheumatology* online.

#### Reliability analysis

The intra-observer reliability κ values for joint and tendon US scoring of GS and PD were excellent, with a κ value of 0.83 for joint GS, 0.97 for joint PD, 0.96 for tendon GS and 0.95 for tendon PD. Further details and statistics for individual joint sites and tendon compartments are listed in supplementary Tables S4 and S5, available at *Rheumatology* online.

## Results

### Patient characteristics

One hundred and seven patients were included in the analysis (baseline data are shown in Table 1). As sero-positivity for RF and/or ACPA is a strong predictor of RA, results are presented for both the whole cohort and seronegative patients (defined as patients who were both RF- and ACPA-negative).

**Table 1:** Baseline characteristics for all patients and seronegative patients by diagnostic outcomes

Diagnostic group	Persistent RA (RA)	Non-RA persistent (NRAP)	Resolving (RES)	*P*-value* RA *vs* NRAP	*P*-value* RA *vs* RES
*n* (%)	46 (43)	17 (16)	44 (41)		
Age, years	61 (49–67)	39 (32–64)	44 (33–58)	0.019	0.003
Female, *n* (%)	24 (52)	11 (65)	25 (57)	NA	NA
Symptom duration, weeks	7 (5–9)	5 (4–8)	5 (3–7)	0.175	0.006
Morning stiffness, min	105 (60–180)	60 (10–180)	30 (0–60)	0.393	NS
NSAID use, *n* (%)	33 (72)	13 (76)	27 (61)	NA	NA
RF positivity, *n* (%)	22 (48)	2 (12)	3 (7)	0.010	<0.001
ACPA positivity, *n* (%)	20 (43)	1 (6)	3 (7)	0.006	<0.001
ESR, mm/h	24 (12–39)	32 (11–59)	18 (5–32)	NA	NA
CRP, mg/l	13 (5–34)	24 (9–39)	10 (1–27)	NA	NA
Swollen joint count of 66	7 (3–14)	2 (1–7)	2 (1–5)	0.002	<0.001
Tender joint count of 68	11 (4–15)	5 (2–12)	4 (1–7)	0.123	0.002
Presence of X-ray erosion^a^	1/46 (2.2)	1/16 (6.3)	1/39 (2.6)	NA	NA

**Diagnostic group**	**Seronegative persistent RA (RA)**	**Seronegative non-RA persistent (NRAP)**	**Seronegative resolving RES)**	***P*-value RA *vs* NRAP**	***P*-value RA *vs* RES**
*n* (%)	23 (30)	14 (18)	39 (51)		
Age, years	60 (49–69)	39 (32–72)	43 (33–55)	0.84	0.023
Female, *n* (%)	11 (48)	9 (64)	22 (56)	NA	NA
Symptom duration, weeks	7 (5–9)	6 (4–8)	5 (3–7)	0.923	0.023
Morning stiffness, min	120 (60–240)	60 (8–180)	30 (5–60)	0.359	0.001
NSAID use, *n* (%)	15 (65)	10 (71)	25 (64)	NA	NA
ESR, mm/h	19 (7–47)	36 (12–55)	18 (5–36)	NA	NA
CRP, mg/l	12 (0–26)	21 (5–36)	10 (1–29)	NA	NA
Swollen joint count of 66	7 (3–11)	2 (1–6)	2 (1–5)	0.005	0.001
Tender joint count of 68	12 (4–15)	5 (2–10)	5 (2–7)	0.093	0.027

All variables are shown as median (interquartile range) unless otherwise specified.

a A total of 101 out of 107 patients had hand and/or foot X-ray.

*If the *P*-values for the comparison across the three groups is >0.05, the *P*-values of RA *vs* NRAP and RA *vs* RES is not calculated (NA).

Forty-six patients (43%) developed persistent RA (referred to as RA hereafter), 17 patients (16%) developed non-RA persistent inflammatory arthritis and the remaining 44 (41%) had a resolving disease course, including 10 patients who fulfilled the 2010 ACR/EULAR criteria for RA during the study period but whose disease had resolved by 18 months of follow-up. Only two patients in this sub-group had received DMARD therapy (which was subsequently withdrawn). Further details of these patients are shown in supplementary Table S6, available at *Rheumatology* online. Final diagnoses of patients within the non-RA persistent inflammatory arthritis and resolving groups are listed in supplementary Tables S6 and S7, available at *Rheumatology* online. Of the 46 persistent RA patients, 23 were seronegative.

### Distribution of US-defined joint synovitis

**Fig. 1:**
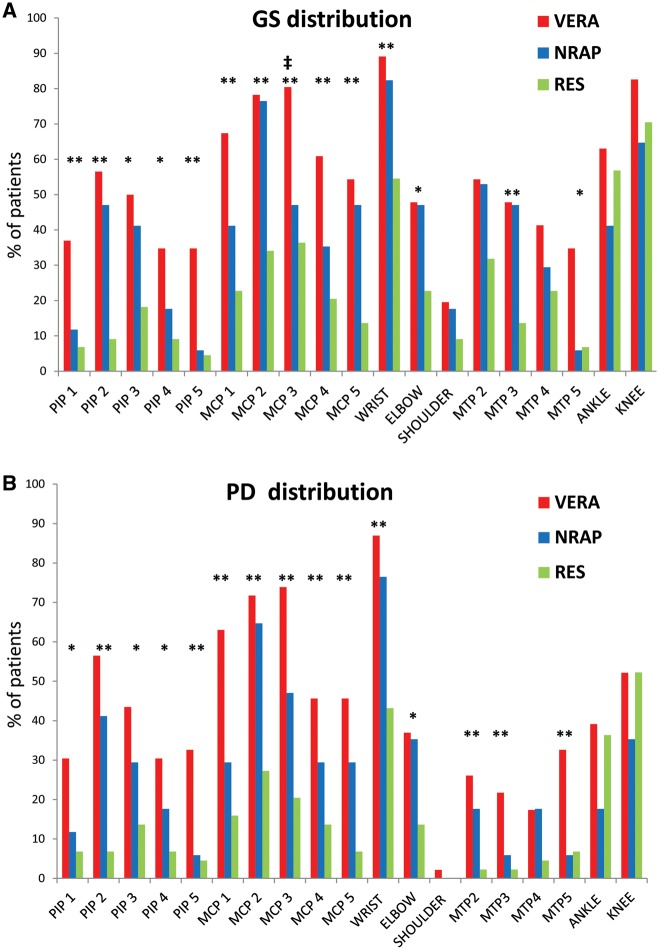
Distribution of joint US pathology in all patients Each bar represents the proportion of patients with US-defined joint synovitis involvement defined by (**A**) Greyscale synovial hypertrophy and (**B**) Power Doppler enhancement. *P* ≤ 0.017 (i.e. 0.05/3) was considered statistically significant as we adjusted for multiple comparisons using the Bonferroni method. VERA *vs* NRAP: ^‡^*P* < 0.001. VERA v*s* RES: *P < 0.017, **P < 0.001. VERA: very early RA; NRAP: non-RA persistent inflammatory arthritis; RES: resolving disease.

A total of 4066 joints (i.e. 19 bilateral joints in 107 patients) were included in the analysis. The distribution of US-defined joint synovitis is presented in Fig. 1. Compared with patients with resolving arthritis, RA patients were more likely to have GS and PD changes at PIP 1–5, MCP 1–5, wrist, elbow, MTP 3 and MTP 5 joints. In addition, RA patients were more likely to have MTP 2 PD changes, but not GS changes alone, compared with patients with resolving arthritis. The only US synovitis variable discriminative of RA from all non-RA patients was MCP 3 GS joint changes (Fig. 1A).

The distribution of US-defined joint synovitis for seronegative patients is presented in supplementary Fig. S1, available at *Rheumatology* online. Compared with patients with resolving arthritis, seronegative RA patients were more likely to have GS changes at the PIP 2, MCP 1, 2, 4 and 5 joints and PD changes at the PIP 2, 3, MCP 1, 2, 3, 5, wrist and MTP 2 joints.

### Distribution of US-defined TS

**Fig. 2:**
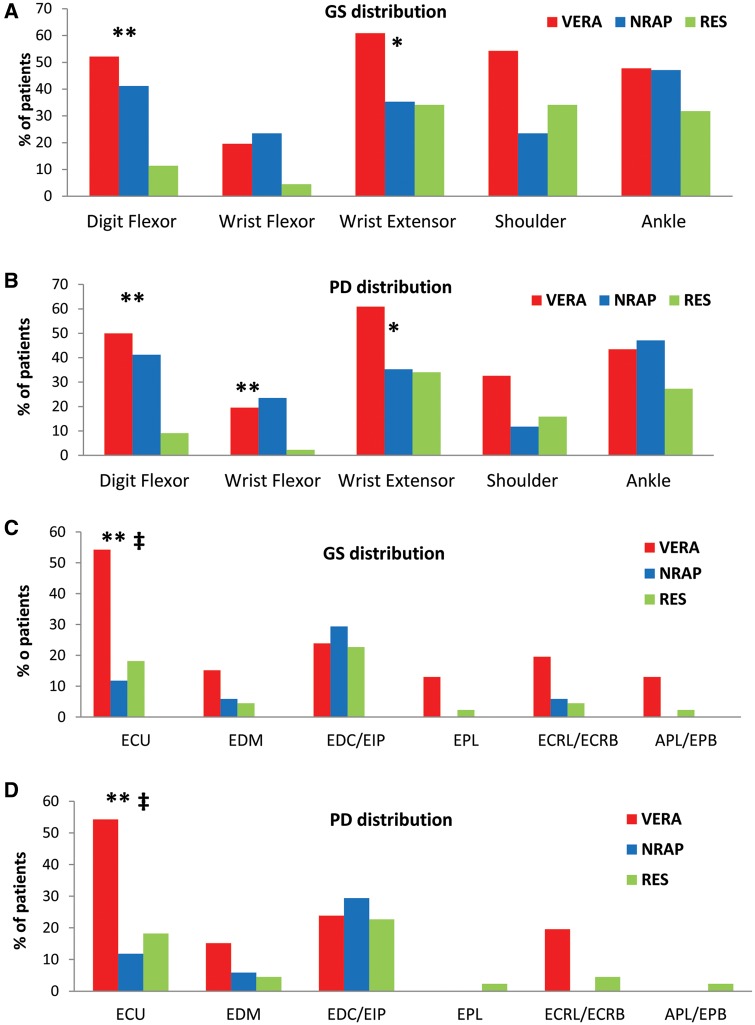
Distribution of tendon US pathology in all patients Each bar represents the proportion of patients US-defined tenosynovitis involvement according to (**A**) and (**B**) tendon regions, (**C**) and (**D**) wrist extensor compartments. *P* < 0.017 (i.e. 0.05/3) was considered statistically significant as we adjusted for multiple comparisons using the Bonferroni method. VERA *vs* NRAP: ^‡^*P* < 0.001. VERA *vs* RES *P < 0.017, **P < 0.001. APL: abductor pollicis longus; EPB: extensor pollcis brevis; ECRL: extensor carpi radialis longus; ECRB: extensor carpi radialis brevis; EPL: extensor pollicis longus; EDC: extensor digitorum communis; EIP: extensor indicis propius; EDM: extensor digiti minimi; ECU: extensor carpi ulnaris; VERA: very early RA; NRAP: non-RA persistent inflammatory arthritis; RES: resolving disease.

Some 3424 tendon compartments (i.e. 16 bilateral tendon compartments in 107 patients) were included in the analysis. All patient groups had evidence of US-defined TS of at least one anatomical site at baseline (RA 85%, non-RA persistent disease 71% and resolving 70%). The distribution of US-defined TS by tendon region for all patients is presented in Fig. 2. Compared with patients with resolving arthritis, RA patients were more likely to have digit flexor and wrist extensor US-defined TS, with both GS and PD pathology. US-detected disease across the six wrist extensor compartments is presented in Fig. 2C and D. Among the wrist extensor tendon compartments, US-defined extensor carpi ulnaris (ECU) TS was more prevalent in RA patients compared with both patients with resolving arthritis and non-RA patients. This was true for both GS and PD.

The distribution of US-defined TS by tendon region for seronegative patients is presented in supplementary Figs S2 and S3, available at *Rheumatology* online. US-defined digit flexor GS and PD TS were more prevalent in the RA group compared with the resolving arthritis group (supplementary Fig. S2, available at *Rheumatology* online).

### Predictive value of clinical, serological and US variables

#### Univariate analysis

*Clinical and serological predictors of RA*. Univariate logistic regression analysis was performed with the clinical, serological and US predictors as independent variables, and RA *vs* non-RA outcome at 18 months as the dependent variable.

The clinical, serological and US predictors of RA for all patients on univariate analysis are shown in Table 2. Age ⩾60 years, early morning stiffness of duration ⩾60 min, swollen joint count-66 and tender joint count-68 ⩾6 and symptom duration ⩾6 weeks were predictors of RA on univariate analysis. The remaining clinical and serological variables were not predictive of RA on univariate analysis (supplementary Table S8, available at *Rheumatology* online). In seronegative patients, only age ⩾60 years, early morning stiffness of duration ⩾60 min and swollen joint count-66 ⩾6 were predictors of seronegative RA (supplementary Table S9, available at *Rheumatology* online).

**Table 2:** Univariate analysis of clinical, serological and US variables at baseline for all patients in the prediction of RA

	OR (95% CI)	*P*-value	RA patients, *n* (%) (*n* = 46)	Non-RA patients, *n* (%) (*n* = 61)
Clinical variables				
Age ≥60 years	3.662 (1.595, 8.408)	0.002	24 (52)	14 (23)
Swollen joint count-66 ≥6 joints	3.662 (1.595, 8.408)	0.002	24 (52)	14 (23)
Tender joint count-68 ≥6 joints	2.456 (1.119, 5.394)	0.025	29 (63)	25 (41)
Early morning stiffness duration ≥60 min	3.972 (1.677, 9.408)	0.002	36 (78)	29 (48)
Symptom duration ≥6 weeks	2.878 (1.286, 6.445)	0.010	32 (70)	27 (44)
Serological variables				
ACPA positivity^a^	10.962 (3.404, 35.298)	0.000	20 (43)	4 (7)
ACPA high-positivity^b^	9.161 (2.832, 29.635)	0.000	18 (39)	4 (7)
RF positivity^c^	10.267 (3.478, 30.304)	0.000	22 (48)	5 (8)
RF high-positivity^d^	17.293 (3.740, 79.951)	0.000	17 (37)	2 (3)
US variables				
Joint US variables^e^				
MCP 1 GS	5.349 (2.326, 12.299)	0.000	31 (67)	17 (28)
MCP 1 PD	6.966 (2.918, 16.627)	0.000	29 (63)	12 (20)
MCP 2 GS	4.243 (1.790, 10.055)	0.001	36 (78)	28 (46)
MCP 2 PD	4.194 (1.839, 9.567)	0.001	33 (72)	23 (38)
MCP 3 GS	6.338 (2.599, 15.455)	0.000	37 (80)	24 (39)
MCP 3 PD	7.333 (3.091, 17.398)	0.000	34 (74)	17 (28)
MCP 4 GS	4.770 (2.078, 10.949)	0.000	28 (61)	15 (25)
MCP 4 PD	3.818 (1.594, 9.144)	0.003	21 (46)	11 (18)
MCP 5 GS	3.997 (1.739, 9.186)	0.001	25 (54)	14(23)
MCP 5 PD	5.565 (2.167, 14.289)	0.000	21 (46)	8 (13)
PIP 1 GS	6.566 (2.200, 19.592)	0.001	17 (37)	5 (8)
PIP 1 PD	4.900 (1.615, 14.863)	0.005	14 (30)	5 (8)
PIP 2 GS	5.308 (2.248, 12.535)	0.000	26 (57)	12 (20)
PIP 2 PD	6.630 (2.712, 16.210)	0.000	26 (57)	10 (16)
PIP 3 GS	3.067 (1.350, 6.968)	0.007	23 (50)	15 (25)
PIP 3 PD	3.497 (1.457, 8.389)	0.005	20 (43)	11 (18)
PIP 4 GS	4.114 (1.523, 11.117)	0.005	16 (35)	7 (11)
PIP 4 PD	4.010 (1.402, 11.471)	0.010	14 (30)	6 (10)
PIP 5 GS	10.311 (2.783, 38.197)	0.000	16 (35)	3 (5)
PIP 5 PD	9.355 (2.514, 34.811)	0.001	15 (33)	3 (5)
Elbow GS	2.190 (0.986, 4.866)	0.054	22(48)	18 (30)
Elbow PD	2.394 (1.003, 5.714)	0.049	17 (37)	12 (20)
Wrist GS	4.963 (1.714, 14.369)	0.003	41 (89)	38 (62)
Wrist PD	6.042 (2.235, 16.331)	0.000	40 (87)	32 (52)
MTP 2 GS	1.967 (0.904, 4.280)	0.088	25 (54)	23 (38)
MTP 2 PD	5.029 (1.502, 16.844)	0.009	12 (26)	4 (7)
MTP 3 GS	3.077 (1.340, 7.065)	0.008	22 (48)	14 (23)
MTP 3 PD	8.194 (1.698, 39.536)	0.009	10 (22)	2 (3)
MTP 5 GS	7.600 (2.332, 24.770)	0.001	16(35)	4 (7)
MTP 5 PD	6.895 (2.105, 22.586)	0.001	15 (33)	4 (7)
Tendon US variable				
Wrist ECU GS	6.071 (2.488, 14.818)	0.000	25 (54)	10 (16)
Wrist ECU PD	6.071 (2.488, 14.818)	0.000	25 (54)	10 (16)
Digit flexor GS	4.455 (1.892, 10.488)	0.001	24 (52)	12 (20)
Digit flexor PD	4.545 (1.901, 10.869)	0.001	23 (50)	11 (18)
Wrist extensor GS	2.963 (1.340, 6.551)	0.007	28 (61)	21 (34)
Wrist extensor PD	2.963 (1.340, 6.551)	0.007	28 (61)	21 (34)
Shoulder biceps GS	3.345 (1.472, 7.605)	0.004	24 (52)	15 (25)
Shoulder biceps PD	2.796 (1.094, 7.146	0.032	15 (33)	9 (15)

a ACPA >7 IU/ml.

b ACPA >21 IU/ml.

c RF >20 IU/ml.

d RF >60 IU/ml.

e GS grading ≥1; PD grading ≥1; US pathology was present in at least unilateral joint. ECU: extensor carpi ulnaris tendon; GS: grey scale; OR: odds ratio; PD: power Doppler.

*US-defined joint synovitis predictors of RA*. GS and PD US synovitis of the MCP 1–5, PIP 1–5, wrist, MTP 3 and MTP 5 joints were predictors of RA (Table 2). In addition, MTP 2 PD joint synovitis, but not GS synovitis alone, was a predictor of RA.

The GS joint US variables predictive of seronegative RA were MCP 1, 3, 4, 5, PIP 1, 5 and MTP 5. The PD joint US variables predictive of seronegative RA were MCP 1, 3, 5, PIP 1, 2, 3, 4 and MTP 2. US-defined joint synovitis variables that were not predictive of RA and seronegative RA are shown in supplementary Tables S10 and S11, available at *Rheumatology* online, respectively.

*US-defined TS predictors of RA*. US-defined digit flexor and wrist ECU were predictive of RA. The predictive abilities of GS and PD variables for each tendon compartment were comparable (Table 2). The predictive values of other tendon compartments are listed in supplementary Table S12, available at *Rheumatology* online. For seronegative patients, digit flexor and ECU remained as predictors of seronegative RA. The predictive ability of GS and PD for each tendon compartment was also comparable (supplementary Table S13, available at *Rheumatology* online).

#### PCA

In this step, statistically significant variables from the univariate analysis were included in PCA analyses in order to identify the key variables that would account for the majority of the explanatory variance observed. In particular, we wished to test the hypothesis that US-measured joint and tendon variables would cluster in separate components, indicating non-correlation.

Two PCA analyses were performed, one for US and one for clinical and serological variables. The number of components extracted was based on eigenvalues with a cut-off of one and the rotation method adopted was according to the varimax criteria with Kaiser normalization.

The rotated factor loadings of the PCA for each clinical, serological and US variable are shown in supplementary Tables S14 and S15, available at *Rheumatology* online. Three components were extracted from the clinical and serological PCA, whilst nine components were extracted from the joint and tendon US PCA (Table 3).

**Table 3:** Components from the clinical, serological and US PCA

**PCA of clinical and serological variables**										
**Components**	**1**				**2**			**3**		
Variables	ACPA positivity ACPA high-positivity RF positivity RF high-positivity				Swollen joint count-66 ≥6 Tender joint count-68 ≥6 Early morning stiffness duration ≥60 min			Symptom duration ≥6 weeks Age ≥60 years old		
% of variance explained	38.25				17.87			12.30		
Cumulative % of variance explained **=** 68.41										

**PCA of US variables**										
**Components**	**1**	**2**	**3**	**4**		**5**	**6**	**7**	**8**	**9**
Variables	MCP 1 MCP 2 MCP 3 MCP 4	PIP 2 PIP 3 PIP 4 PIP 5	PIP 1 PIP 4	Digit flexor		MTP 2 MTP 3	ECU Shoulder tendon	MTP 5	Wrist joint	MCP 5
% of variance explained	38.01	8.54	7.21	5.84		5.29	4.26	3.97	3.69	3.12
Cumulative % of variance explained = 79.93										

ECU: extensor carpi ulnaris tendon; PCA: principal component analyses.

### Multivariate logistic regression

Subsequently, a multiple logistic regression model was developed using the variables identified by PCA. The variable with the highest loading factor from each component was extracted and made available as an independent variable in a forward step-wise multivariate logistic regression analysis, with RA outcome at 18 months entered as the dependent variable. These variables are listed in supplementary Table S16, available at *Rheumatology* online. The logistic regression analysis identified PIP1 PD, digit flexor GS and ACPA positivity as the variables which formed the model for the prediction of RA, with the proportion of RA *vs* non-RA identified as 75.7% (supplementary Table S17, available at *Rheumatology* online). In order to robustly confirm that US-measured joint and tendon variables provided independent predictive value, a further regression analysis was performed (supplementary Table S17, available at *Rheumatology* online). In this case, we systematically entered US joint variables identified in the univariate analysis.

The optimal combination identified was MCP 3 PD, digit flexor GS and ACPA positivity (Table 4), with the proportion of RA *vs* non-RA patients correctly identified in our cohort being 80.4%. Removing the digit flexor variable in this regression model results in the proportion of RA *vs* non-RA correctly identified falling from 80.4 to 73.8%.

**Table 4:** Logistic regression model

Variable	OR	95% CI	*P*-value	Nagelkerke *R*^2^	% of patients correctly identified (RA *vs* non-RA)
ACPA positivity	10.973	3.031–39.730	0.000	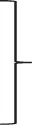 0.439	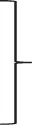 80.4
MCP 3 PD positivity	4.066	1.444–11.444	0.008		
Digit flexor tendon GS	3.078	1.047–9.046	0.041		
ACPA positivity	9.324	2.648–32.832	0.001	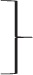 0.402	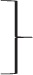 73.8
MCP 3 PD positivity	6.451	2.525–16.482	0.000		

OR: odds ratio.

## Discussion

Previous studies have reported that US-defined joint synovitis improves the prediction of RA above and beyond clinical and serological variables in early arthritis patients [3] and also improves the prediction of RA in seronegative unclassified arthritis patients [18].

In this study, we showed that US-defined TS, specifically digit flexor TS, provides additional predictive data alongside US-defined joint synovitis and other clinical and serological variables in a cohort of patients with early arthritis.

These findings are consistent with studies of gadolinium-enhanced MRI, in which digit flexor TS was a significant predictor of early RA in a cohort of patients with undifferentiated arthritis or clinically suspected RA with no joint swelling [19]. In agreement with our data, the authors concluded that MRI-defined digit flexor TS provided additional predictive data for patients in their cohort even in the presence of ACPA or RF. In addition, longitudinal data from the Leiden Early Arthritis Clinic showed that MRI-defined TS of the fifth ray flexor tendons was more common in early arthritis patients who later developed RA compared with those who did not [7].

Grassi *et al.* first described sonographic changes affecting the hand flexor tendon in RA patients. The authors reported that 90% (18/20) of RA patients had sonographic changes at either digit flexor and/or extensor tendons [20]. Subsequent US studies described the distribution of tendon involvement in the hands and/or wrists of RA patients [21, 22]. The present study is the first to describe the distribution of US-defined TS of multiple tendon sites, including the shoulder and ankle regions, in early arthritis. In addition, this study includes the most extensive US assessment to date, including the MCP, PIP, wrist, MTP, knee, ankle and elbow joints, and digit, wrist, shoulder and ankle tendons.

One of the main challenges in US studies is identifying the minimal joint, or tendon, subset that will provide the maximal predictive ability for a given outcome [23]. We performed a PCA of joint and tendon US variables to identify redundant US variables within a given patient group. Importantly, in this study we showed that tendon US variables were not redundant with their corresponding regional joint US variables. For example, digit flexor tendon US variables were not placed within the same component as small joint synovitis variables of MCP or PIP joints. Similarly, wrist ECU tendon involvement did not share the same component as the wrist joint US variable. These key findings, which are reported for the first time in an early arthritis US study, suggest that tendon US variables provide additional predictive value alongside joint US variables in the context of early arthritis.

One of the strengths of our study is that it was undertaken prospectively in a real world setting. Consecutive patients were recruited from well-established rheumatology centres in the UK that had a wide catchment area. Patients from our cohort also had very short symptom duration, with median symptom duration of between 5 and 7 weeks. These findings suggest that US-detected TS alongside US-detected joint synovitis is a reliable imaging biomarker in the very early phase of arthritis, falling within the proposed 12-week therapeutic window of opportunity of early arthritis [24].

Whilst several studies have assessed the tenosynovium in patients with RA compared with healthy controls [6, 20], an additional strength of our study is that we assessed the predictive utility of TS assessment in a clinically meaningful context of an unselected early arthritis cohort. The comparator groups are patients with resolving and non-RA disease—patients frequently seen in early arthritis clinics and in relation to which management decisions have to be made on the basis of prediction of future outcomes.

The main limitation of our study relates to the relatively small size of this initial cohort, necessitated by the extensive imaging performed per patient. A larger sample size is required in order to design weighted predictive algorithms and identify specific domains such as individual flexor tendons that provide the most useful predictive data in order to reduce scanning time.

Previous imaging studies illustrated that gadolinium-enhanced MRI-defined digit flexor TS is an independent predictor of RA. Our findings demonstrate similar findings for US, a more accessible point-of-care imaging tool. Our data show that US-defined digit flexor TS provides independent predictive value for RA development in early arthritis patients. This finding should be further evaluated in a larger study, and investigators testing imaging-based variables within predictive algorithms for RA development should consider including this tendon component as a candidate variable.

## Supplementary Material

Supplementary Data

## References

[1] van der Linden MP, le Cessie S, Raza K et al Long-term impact of delay in assessment of patients with early arthritis. Arthritis Rheum 2010;62:3537–46. 20722031 10.1002/art.27692

[2] van der Helm-van Mil AH, Detert J, le Cessie S et al Validation of a prediction rule for disease outcome in patients with recent-onset undifferentiated arthritis: moving towards individualized treatment decision-making. Arthritis Rheum 2008;58:2241–7. 18668546 10.1002/art.23681

[3] Filer A, de Pablo P, Allen G et al Utility of ultrasound joint counts in the prediction of rheumatoid arthritis in patients with very early synovitis. Ann Rheum Dis 2011;70:500–7. 21115552 10.1136/ard.2010.131573 PMC3033529

[4] Nakagomi D, Ikeda K, Okubo A et al Ultrasound can improve the accuracy of the 2010 American College of Rheumatology/European League against rheumatism classification criteria for rheumatoid arthritis to predict the requirement for methotrexate treatment. Arthritis Rheum 2013;65:890–8. 23334942 10.1002/art.37848

[5] Wakefield RJ, Green MJ, Marzo-Ortega H et al Should oligoarthritis be reclassified? Ultrasound reveals a high prevalence of subclinical disease. Ann Rheum Dis 2004;63:382–5. 15020331 10.1136/ard.2003.007062 PMC1754934

[6] Wakefield RJ, O’Connor PJ, Conaghan PG et al Finger tendon disease in untreated early rheumatoid arthritis: a comparison of ultrasound and magnetic resonance imaging. Arthritis Rheum 2007;57:1158–64. 17907233 10.1002/art.23016

[7] Nieuwenhuis WP, Krabben A, Stomp W et al Evaluation of magnetic resonance imaging-detected tenosynovitis in the hand and wrist in early arthritis. Arthritis Rheumatol 2015;67:869–76. 25510520 10.1002/art.39000

[8] Filippucci E, Gabba A, Di Geso L et al Hand tendon involvement in rheumatoid arthritis: an ultrasound study. Semin Arthritis Rheum 2012;41:752–60. 22055542 10.1016/j.semarthrit.2011.09.006

[9] Kaibara N, Yamada H, Shuto T et al Comparative histopathological analysis between tenosynovitis and joint synovitis in rheumatoid arthritis. Histopathology 2008;52:856–64. 18462359 10.1111/j.1365-2559.2008.03050.x

[10] Bruyn GA, Moller I, Garrido J et al Reliability testing of tendon disease using two different scanning methods in patients with rheumatoid arthritis. Rheumatology (Oxford) 2012;51:1655–61. 22627728 10.1093/rheumatology/kes103

[11] Naredo E, D’Agostino MA, Wakefield RJ et al Reliability of a consensus-based ultrasound score for tenosynovitis in rheumatoid arthritis. Ann Rheum Dis 2013;72:1328–34. 22984169 10.1136/annrheumdis-2012-202092

[12] Aletaha D, Neogi T, Silman AJ et al 2010 Rheumatoid arthritis classification criteria: an American College of Rheumatology/European League Against Rheumatism collaborative initiative. Arthritis Rheum 2010;62:2569–81. 20872595 10.1002/art.27584

[13] Kingsley G, Sieper J. Third International Workshop on Reactive Arthritis. 23-26 September 1995, Berlin, Germany. Report and abstracts. Ann Rheum Dis 1996;55:564–84. 8815821 10.1136/ard.55.8.564 PMC1010245

[14] Rudwaleit M, van der Heijde D, Landewe R et al The Assessment of SpondyloArthritis International Society classification criteria for peripheral spondyloarthritis and for spondyloarthritis in general. Ann Rheum Dis 2011;70:25–31. 21109520 10.1136/ard.2010.133645

[15] Taylor W, Gladman D, Helliwell P et al Classification criteria for psoriatic arthritis: development of new criteria from a large international study. Arthritis Rheum 2006;54:2665–73. 16871531 10.1002/art.21972

[16] Szkudlarek M, Klarlund M, Narvestad E et al Ultrasonography of the metacarpophalangeal and proximal interphalangeal joints in rheumatoid arthritis: a comparison with magnetic resonance imaging, conventional radiography and clinical examination. Arthritis Res Ther 2006;8:R52. 16519793 10.1186/ar1904 PMC1526591

[17] Schmidt WA, Schmidt H, Schicke B, Gromnica-Ihle E. Standard reference values for musculoskeletal ultrasonography. Ann Rheum Dis 2004;63:988–94. 15249327 10.1136/ard.2003.015081 PMC1755091

[18] Freeston JE, Wakefield RJ, Conaghan PG et al A diagnostic algorithm for persistence of very early inflammatory arthritis: the utility of power Doppler ultrasound when added to conventional assessment tools. Ann Rheum Dis 2010;69:417–9. 19359260 10.1136/ard.2008.106658

[19] Eshed I, Feist E, Althoff CE et al Tenosynovitis of the flexor tendons of the hand detected by MRI: an early indicator of rheumatoid arthritis. Rheumatology (Oxford) 2009;48:887–91. 19474128 10.1093/rheumatology/kep136

[20] Grassi W, Tittarelli E, Blasetti P, Pirani O, Cervini C. Finger tendon involvement in rheumatoid arthritis. Evaluation with high-frequency sonography. Arthritis Rheum 1995;38:786–94. 7779121 10.1002/art.1780380611

[21] Backhaus M, Kamradt T, Sandrock D et al Arthritis of the finger joints: a comprehensive approach comparing conventional radiography, scintigraphy, ultrasound, and contrast-enhanced magnetic resonance imaging. Arthritis Rheum 1999;42:1232–45. 10366117 10.1002/1529-0131(199906)42:6<1232::AID-ANR21>3.0.CO;2-3

[22] Hoving JL, Buchbinder R, Hall S et al A comparison of magnetic resonance imaging, sonography, and radiography of the hand in patients with early rheumatoid arthritis. J Rheumatol 2004;31:663–75. 15088290

[23] D'Agostino MA, Terslev L, Wakefield R et al Novel algorithms for the pragmatic use of ultrasound in the management of patients with rheumatoid arthritis: from diagnosis to remission. Ann Rheum Dis 2016;75:1902–8. 27553213 10.1136/annrheumdis-2016-209646

[24] van Nies JA, Krabben A, Schoones JW et al What is the evidence for the presence of a therapeutic window of opportunity in rheumatoid arthritis? A systematic literature review. Ann Rheum Dis 2014;73:861–70. 23572339 10.1136/annrheumdis-2012-203130

