# Supplementary material for: The role of ultrasound-defined tenosynovitis and synovitis in the prediction of rheumatoid arthritis development
Source: Rheumatology (Oxford). 2018 Apr 3;57(7):1243–52. doi: 10.1093/rheumatology/key025 (PMC6037116; doi:10.1093/rheumatology/key025)
Supplement: Supplementary Data [file key025_suppl_data.docx]

# SUPPLEMENTARY DATA

# Supplementary Table S1. Synovial intra-articular recesses and periarticular sites evaluated by ultrasound

| **Joint*** | **Recess** |
| --- | --- |
| **MCP (1-5), PIP (1-5), MTP (2-5)** | Multi-planar scanning of dorsal recesses |
|  | Lateral recess of MCP1,2,MTP5 |
| **Wrist** | Intercarpal recesses |
|  | Radiocarpal recesses |
|  | Ulnarcarpal recesses |
|  | Volar carpal recesses |
| **Elbow** | Anterior recess |
|  | Humeroradial joint |
|  | Humeroulnar joint |
|  | Posterior recess |
| **Shoulder** | Posterior glenohumeral recess |
| **Knee** | Suprapatellar recess |
|  | Medial parapatellar recess |
|  | Lateral parapatellar recess |
|  | Medial femorotibial joint line |
|  | Lateral femorotibial joint line |
| **Ankle** | Anterior tibiotalar recess |
|  | Medial tibiotalar recess |
|  | Lateral tibiotalar recess |

MCP: metacarpophalangeal joint; MTP: metatarsophalangeal joint; PIP: proximal interphalangeal joint.

# Supplementary Table S2 Tendon compartments evaluated by ultrasound

| Tendon | Tendon Compartment |
| --- | --- |
| Digit | Flexor tendon 1-5 |
| Wrist | Flexor tendon compartment |
|  | Extensor compartment 1 (APL and EPB) |
|  | Extensor compartment 2 (ECRL and ECRB) |
|  | Extensor compartment 3 (EPL) |
|  | Extensor compartment 4 (EDC and EIP) |
|  | Extensor compartment 5 (EDM) |
|  | Extensor compartment 6 (ECU) |
| Shoulder | Biceps tendon |
| Ankle | Anterior extensor compartment (TA, EHL, EDL) |
|  | Peroneus longus and brevis |
|  | Posteromedial compartment (PT, FDL, FHL) |

APL: Abductor pollicis longus; EPB: extensor pollicis brevis; ECRL: extensor carpi radialis longus; ECRB: Extensor carpi radialis brevis; EPL: Extensor pollicis longus; EDC: extensor digitorum communis; EIP: Extensor indicis propius; EDM: Extensor digiti minimi; ECU: extensor carpi ulnaris; TA: Tibialis Anterior; EHL: Extensor Hallucis Longus; EDL: Extensor digitorum Longus; PT: Posterior tibial; FDL: Flexor digitorum longus FHL: Flexor hallucis longus.

# Supplementary Table S3 Classification of variables for the logistic regression analysis

| **Variables** | **Category** |
| --- | --- |
| Gender | Male*/female |
| Age | < 60 years*/ ≥60 years |
| ESR | Normal*/abnormal (by local standards) |
| CRP | Normal*/abnormal (by local standards) |
| Swollen joint count-66 | 1*, 2–5, 6–66 |
| Tender joint count-68 | 1*, 2–5, 6–68 |
| Early morning stiffness | <60 minutes* / ≥60 minutes |
| Rheumatoid factor (RF) | Normal* / low-positive^a^  Normal* / high-positive^b^ |
| Anti-cyclic citrullinated peptide (ACPA) | Normal* / low-positive^c^  Normal* / high-positive^d^ |

*Reference category. ^a^ RF > 20 IU/mL, ^b^ RF > 60 IU/mL, ^c^ACPA >7 EU/ml, ^d^ACPA >21 EU/ml.

# Supplementary Table S4. Intra-observer reliability of joint ultrasound assessment.

| **Joint assessment** | **Greyscale** | **Power Doppler** |
| --- | --- | --- |
| **Overall** | **0.83** | **0.87** |
| PIP | 0.85 | 0.91 |
| MCP | 0.85 | 0.91 |
| Wrist | 0.77 | 0.81 |
| Elbow | 0.81 | 0.82 |
| Shoulder | 0.86 | 0.65 |
| Knee | 0.77 | 0.70 |
| Ankle | 0.77 | 0.75 |
| MTP | 0.76 | 0.88 |

A kappa value of 0-0.2 was considered poor, 0.21-0.40 fair, 0.41-0.6 moderate, 0.61-0.8 good, and 0.81 to 1 excellent. Intra-observer reliability was evaluated by blindly rescoring representative images of 20 patients for joint ultrasound assessments and analysed using κ statistics. MCP: metacarpophalangeal joint; PIP: proximal interphalangeal joint.

# Supplementary Table S5. Intra-observer reliability of tendon ultrasound assessment.

| Tendon assessment | Greyscale | Power Doppler |
| --- | --- | --- |
| Overall | **0.96** | **0.95** |
| **Digit** | 0.99 | 0.99 |
| **Wrist** | 0.97 | 0.95 |
| **Shoulder** | 0.94 | 0.86 |
| **Ankle** | 0.96 | 0.95 |
| **Tendon Compartments** | **Greyscale** | **Power Doppler** |
| **Digit flexor** | 0.99 | 0.99 |
| **Wrist flexor** | 0.97 | 0.97 |
| **Wrist extensor** | 0.97 | 0.94 |
| **Shoulder** | 0.94 | 0.86 |
| **Ankle anterior** | 0.97 | 0.96 |
| **Ankle posteromedial** | 0.99 | 0.99 |
| **Ankle peroneal** | 0.88 | 0.88 |

Intra-observer reliability was evaluated by blindly rescoring representative images of 20 patients for tendon ultrasound assessments, and analysed using κ statistics.

# Supplementary Table S6. Final diagnoses for all patients

*Of these 10 patients, six patients did not receive any DMARDs or corticosteroids; one patient received a single dose of intra-muscular methylprednisolone; one patient received a short course of prednisolone; one patient received a short course of prednisolone and hydroxychloroquine for three weeks; one patient received methotrexate monotherapy and hydroxychloroquine was added after two months, both DMARDs were withdrawn after six months.

| Final diagnosis,  n | RA  46 | Non-RA Persistent  17 | Resolving  44 |
| --- | --- | --- | --- |
| RA | 46 | 0 | 10* |
| PsA | 0 | 7 | 2 |
| SLE | 0 | 3 | 0 |
| AS | 0 | 1 | 0 |
| Crystal arthropathy | 0 | 0 | 4 |
| Parvovirus arthropathy | 0 | 0 | 4 |
| Post-streptococcal | 0 | 0 | 1 |
| Reactive arthritis | 0 | 1 | 1 |
| Infectious arthritis | 0 | 0 | 1 |
| Sarcoidosis | 0 | 2 | 0 |
| Unclassified | 0 | 3 | 21 |

# Supplementary Table S7. Final diagnoses for seronegative patients (negative for RF and ACPA)

| sw | RA  23 | Non-RA Persistent  14 | Resolving  39 |
| --- | --- | --- | --- |
| RA | 23 | 0 | 8 |
| PsA | 0 | 5 | 2 |
| SLE | 0 | 3 | 0 |
| AS | 0 | 1 | 0 |
| Crystal arthropathy | 0 | 0 | 3 |
| Parvovirus arthropathy | 0 | 0 | 4 |
| Post-streptococcal | 0 | 0 | 1 |
| Reactive arthritis | 0 | 0 | 1 |
| Infectious arthritis | 0 | 0 | 1 |
| Sarcoidosis | 0 | 2 | 0 |
| Unclassified | 0 | 3 | 19 |

# Supplementary Figure S1. Distribution of joint US pathology in seronegative patients

|  |
| --- |
|  |

Each bar represents the proportion of patients who had US-defined joint synovitis involvement. 0.017 (i.e. 0.05/3) was considered statistically significant as we adjusted for multiple comparisons using the Bonferroni method. VERA vs RES: *p≤0.017, **p≤0.001. PIP: proximal interphalangeal joint. MCP: metacarpophalangeal joint; MTP: metatarsophalangeal joint; VERA: Very Early RA; NRAP: non-RA persistent inflammatory arthritis; RES: Resolving disease.

**Supplementary Figure S2. Distribution of tendon US pathology in seronegative patients**

| **A** |
| --- |
|  |

Each bar represents the proportion of patients who had US-defined tenosynovitis involvement according to tendon regions. P ≤ 0.017 (i.e. 0.05/3) was considered statistically significant as we adjusted for multiple comparisons using the Bonferroni method. VERA vs RES *p≤0.017, **p≤0.001. VERA: Very Early RA; NRAP: non-RA persistent inflammatory arthritis; RES: Resolving disease.

# Supplementary Figure S3. Distribution of wrist tendon US pathology in seronegative patients

|  |
| --- |
|  |
| Each bar represents the proportion of patients who had US-defined tenosynovitis involvement according to wrist extensor compartments. APL: Abductor pollicis longus; EPB: extensor pollcis brevis; ECRL: extensor carpi radialis longus; ECRB: Extensor carpi radialis brevis; EPL: Extensor pollicis longus; EDC: extensor digitorum communis; EIP: Extensor indicis propius; EDM: Extensor digiti minimi; ECU: extensor carpi ulnaris; VERA: Very Early RA; NRAP: non-RA persistent inflammatory arthritis; RES: Resolving disease. |

# Supplementary Table S8. Univariate analyses of clinical and serological variables at baseline for all patients in the prediction of RA.

| Clinical and serological variables | | | |
| --- | --- | --- | --- |
| Clinical variables | **Odds Ratio** | **95% CI** | **p value** |
| Age ≥ 60 years * | 3.662 | 1.595 - 8.408 | **0.002** |
| Age < 60 years | 0.273 | 0.119 - 0.627 | 0.002 |
| Female | 1.320 | 0.610 - 2.855 | 0.481 |
| Tender joint count: 1 joint | 0.091 | 0.011 - 0.726 | 0.024 |
| Swollen joint count: 1 joint | 0.100 | 0.022 - 0.458 | 0.025 |
| Tender joint count: 2-5 joints | 0.897 | 0.393 - 2.046 | 0.796 |
| Swollen joint count: 2-5 joints | 0.969 | 0.448 - 2.095 | 0.936 |
| Swollen joint count: ≥ 6 joints | 3.662 | 1.595 - 8.408 | **0.002** |
| Tender joint count: ≥ 6 joints | 2.456 | 1.119 - 5.394 | **0.025** |
| Early morning stiffness duration ≥ 60 mins | 3.972 | 1.677 - 9.408 | **0.002** |
| Symptom duration ≥ 6 weeks | 2.878 | 1.286 - 6.445 | **0.010** |
| Presence of radiographic erosion | 0.589 | 0.052-6.710 | **0.670** |
| Serological variables | **Odds Ratio** | **95% CI** | **p value** |
| Abnormal CRP | 1.552 | 0.655 - 3.679 | 0.318 |
| Abnormal ESR | 1.341 | 0.601 - 2.991 | 0.474 |
| ACPA positivity | 10.962 | 3.404 - 35.298 | **0.000** |
| ACPA high-positivity | 9.161 | 2.832 - 29.635 | **0.000** |
| RF positivity | 10.267 | 3.478 - 30.304 | **0.000** |
| RF high-positivity | 17.293 | 3.740 - 79.951 | **0.000** |

# Supplementary Table S9. Univariate analyses of clinical and serological variables at baseline for seronegative patients in the prediction of RA

| Clinical and serological variables | | | |
| --- | --- | --- | --- |
| Clinical variables | **OR** | **95% CI** | **p value** |
| Age ≥ 60 years old | 3.727 | 1.316-10.553 | **0.013** |
| Age < 60 years old | 0.268 | 0.095-0.760 | 0.013 |
| Female | 0.651 | 0.243-1.740 | 0.392 |
| Tender joint count: 1 joint | 0.000 | 0.000- | 0.999 |
| Swollen joint count: 1 joint | 0.000 | 0.000- | 0.998 |
| Tender joint count: 2-5 joints | 1.250 | 0.454-3.439 | 0.666 |
| Swollen joint count: 2-5 joints | 1.292 | 0.483-3.455 | 0.610 |
| Tender joint count: ≥ 6 joints | 2.029 | 0.748-5.505 | 0.165 |
| Swollen joint count: ≥ 6 joint | 3.727 | 1.316-10.553 | **0.013** |
| Early morning stiffness duration ≥ 60 mins | 3.738 | 1.210-11.547 | **0.022** |
| Symptom duration ≥ 6 weeks | 2.266 | 0.822-6.247 | 0.114 |
| Serological variables | **OR** | **95% CI** | **p value** |
| Abnormal CRP | 1.338 | 0.448-3.999 | 0.602 |
| Abnormal ESR | 1.048 | 0.376-2.921 | 0.929 |

# Supplementary Table S10. Univariate analysis of joint US variables at baseline for all patients in the prediction of RA

| Joint US variables* | **Odds Ratio** | **95% CI** | **p value** |
| --- | --- | --- | --- |
| MCP 1 GS | 5.349 | 2.326-12.299 | 0.000 |
| MCP 1 PD | 6.966 | 2.918-16.627 | 0.000 |
| MCP 2 GS | 4.243 | 1.790-10.055 | 0.001 |
| MCP 2 PD | 4.194 | 1.839-9.567 | 0.001 |
| MCP 3 GS | 6.338 | 2.599-15.455 | 0.000 |
| MCP 3 PD | 7.333 | 3.091-17.398 | 0.000 |
| MCP 4 GS | 4.770 | 2.078-10.949 | 0.000 |
| MCP 4 PD | 3.818 | 1.594-9.144 | 0.003 |
| MCP 5 GS | 3.997 | 1.739 – 9.186 | 0.001 |
| MCP 5 PD | 5.565 | 2.167 – 14.289 | 0.000 |
| PIP 1 GS | 6.566 | 2.200 - 19.592 | 0.001 |
| PIP 1 PD | 4.900 | 1.615 - 14.863 | 0.005 |
| PIP 2 GS | 5.308 | 2.248- 12.535 | 0.000 |
| PIP 2 PD | 6.630 | 2.712 – 16.210 | 0.000 |
| PIP 3 GS | 3.067 | 1.350 – 6.968 | 0.007 |
| PIP 3 PD | 3.497 | 1.457 – 8.389 | 0.005 |
| PIP 4 GS | 4.114 | 1.523 – 11.117 | 0.005 |
| PIP 4 PD | 4.010 | 1.402-11.471 | 0.010 |
| PIP 5 GS | 10.311 | 2.783-38.197 | 0.000 |
| PIP 5 PD | 9.355 | 2.514-34.811 | 0.001 |
| Wrist GS | 4.963 | 1.714-14.369 | 0.003 |
| Wrist PD | 6.042 | 2.235-16.331 | 0.000 |
| Shoulder GS | 1.876 | 0.642-5.485 | 0.250 |
| Shoulder PD | NA | NA | NA |
| Elbow GS | 2.190 | 0.986-4.866 | 0.054 |
| Elbow PD | 2.394 | 1.003-5.714 | 0.049 |
| Ankle GS | 1.546 | 0.708-3.378 | 0.275 |
| Ankle PD | 1.421 | 0.637-3.171 | 0.391 |
| Knee GS | 2.149 | 0.843-5.476 | 0.109 |
| Knee PD | 1.204 | 0.559 – 2.590 | 0.635 |
| MTP 2 GS | 1.967 | 0.904-4.280 | 0.088 |
| MTP 2 PD | 5.029 | 1.502-16.844 | 0.009 |
| MTP 3 GS | 3.077 | 1.340-7.065 | 0.008 |
| MTP 3 PD | 8.194 | 1.698-39.536 | 0.009 |
| MTP 4 GS | 2.158 | 0.944-4.935 | 0.068 |
| MTP 4 PD | 2.358 | 0.717 – 7.757 | 0.158 |
| MTP 5 GS | 7.600 | 2.332-24.770 | 0.001 |
| MTP 5 PD | 6.895 | 2.105-22.586 | 0.001 |

*GS grading ≥ 1; PD grading ≥ 1; US pathology was present in at least unilateral joint. GS: Gray scale; PD: Power Doppler.

# Supplementary Table S11. Univariate analysis of joint US variables at baseline for seronegative patients in the prediction of RA

| Joint US variables* | Odds Ratio | 95% CI | p value |
| --- | --- | --- | --- |
| MCP 1 GS | 3.294 | 1.192 - 9.106 | 0.022 |
| MCP 1 PD | 4.442 | 1.561 - 12.638 | 0.005 |
| MCP 2 GS | 2.762 | 0.976 - 7.813 | 0.056 |
| MCP 2 PD | 2.567 | 0.940 - 7.011 | 0.066 |
| MCP 3 GS | 3.221 | 1.135 - 9.138 | 0.028 |
| MCP 3 PD | 4.336 | 1.534 - 12.259 | 0.006 |
| MCP 4 GS | 3.621 | 1.298 - 10.103 | 0.014 |
| MCP 4 PD | 2.455 | 0.843 - 7.146 | 0.100 |
| MCP 5 GS | 3.132 | 1.106- 8.868 | 0.032 |
| MCP 5 PD | 4.327 | 1.418 - 13.207 | 0.010 |
| PIP 1 GS | 4.200 | 1.168 - 15.099 | 0.028 |
| PIP 1 PD | 4.200 | 1.168 15.099 | 0.028 |
| PIP 2 GS | 2.764 | 0.935 - 8.171 | 0.066 |
| PIP 2 PD | 3.143 | 1.044 - 9.465 | 0.042 |
| PIP 3 GS | 2.367 | 0.841 - 6.663 | 0.103 |
| PIP 3 PD | 3.761 | 1.261 - 11.214 | 0.017 |
| PIP 4 GS | 1.825 | 0.512 - 6.503 | 0.353 |
| PIP 4 PD | 1.175 | 0.267 - 5.169 | 0.831 |
| PIP 5 GS | 4.630 | 1.003 - 21.367 | 0.050 |
| PIP 5 PD | 4.630 | 1.003 - 21.367 | 0.050 |
| Wrist GS | 2.362 | 0.761 - 7.339 | 0.137 |
| Wrist PD | 2.728 | 0.931 - 7.996 | 0.067 |
| Shoulder GS | 0.746 | 0.139 - 4.007 | 0.733 |
| Shoulder PD | NA | NA | NA |
| Elbow GS | 1.782 | 0.658 - 4.827 | 0.256 |
| Elbow PD | 2.196 | 0.764 - 6.314 | 0.144 |
| Ankle GS | 1.287 | 0.475 - 3.488 | 0.619 |
| Ankle PD | 1.250 | 0.454 - 3.439 | 0.666 |
| Knee GS | 2.883 | 0.749 - 11.096 | 0.124 |
| Knee PD | 1.108 | 0.415 - 2.953 | 0.838 |
| MTP 2 GS | 1.512 | 0.563 - 4.066 | 0.412 |
| MTP 2 PD | 4.324 | 1.087 - 17.189 | 0.038 |
| MTP 3 GS | 1.346 | 0.454 - 3.990 | 0.592 |
| MTP 3 PD | 3.825 | 0.594 - 24.630 | 0.158 |
| MTP 4 GS | 1.346 | 0.454 - 3.990 | 0.592 |
| MTP 4 PD | 2.021 | 0.489 - 8.345 | 0.331 |
| MTP 5 GS | 4.324 | 1.087 17.189 | 0.038 |
| MTP 5 PD | 3.403 | 0.821 - 14.098 | 0.091 |

*GS grading ≥ 1; PD grading ≥ 1; US pathology was present in at least unilateral joint. GS: Gray scale; PD: Power Doppler.

# Supplementary Table S12. Univariate analysis of tendon compartment TS at baseline for all patients in the prediction of RA

| Tendon Compartment | OR | 95% CI | p |
| --- | --- | --- | --- |
| ECU GS | 6.07 | 2.49-14.82 | 0.000 |
| ECU PD | 6.07 | 2.49-14.82 | 0.000 |
| Digit Flexor GS | 4.46 | 1.89-10.49 | 0.001 |
| Digit Flexor PD | 4.55 | 1.90-10.87 | 0.001 |
| Wrist Extensor GS | 2.27 | 1.04-4.97 | 0.041 |
| Wrist Extensor PD | 2.84 | 1.28-6.33 | 0.010 |
| Wrist Flexor GS | 2.23 | 0.73-6.80 | 0.158 |
| Wrist Flexor PD | 2.72 | 0.85-8.77 | 0.093 |
| Shoulder Biceps GS | 3.35 | 1.47-7.605 | 0.004 |
| Shoulder Biceps PD | 2.80 | 1.09-7.15 | 0.032 |
| Ankle Extensor GS | 0.87 | 0.29-2.64 | 0.801 |
| Ankle Extensor PD | 0.71 | 0.22-2.26 | 0.557 |
| Ankle Posterior Tibialis GS | 1.42 | 0.64-3.17 | 0.391 |
| Ankle Posterior Tibialis PD | 1.50 | 0.65-3.45 | 0.340 |
| Ankle Peroneal GS | 2.42 | 0.86-6.85 | 0.095 |
| Ankle Peroneal PD | 2.42 | 0.86-6.85 | 0.095 |

GS: Gray scale; PD: Power Doppler; TS: tenosynovitis.

# Supplementary Table S13. Univariate of tendon compartment TS at baseline in seronegative patients for the prediction of RA

| Tendon Compartment | OR | 95% CI | p |
| --- | --- | --- | --- |
| ECU GS | 3.76 | 1.26-11.21 | 0.017 |
| ECU PD | 3.76 | 1.26-11.21 | 0.017 |
| Digit Flexor GS | 4.97 | 1.72-14.30 | 0.003 |
| Digit Flexor PD | 4.69 | 1.61-13.66 | 0.005 |
| Wrist Extensor GS | 1.94 | 0.71-5.28 | 0.194 |
| Wrist Extensor PD | 2.55 | 0.92-7.09 | 0.072 |
| Wrist Flexor GS | 2.67 | 0.69-10.32 | 0.155 |
| Wrist Flexor PD | 3.40 | 0.82-14.10 | 0.091 |
| Shoulder Biceps GS | 4.79 | 1.68-13.61 | 0.003 |
| Shoulder Biceps PD | 3.62 | 1.17-11.14 | 0.025 |
| Ankle Extensor GS | 0.536 | 0.11-2.75 | 0.454 |
| Ankle Extensor PD | 0.536 | 0.11-2.75 | 0.454 |
| Ankle Posterior Tibialis GS | 0.642 | 0.20-2.03 | 0.451 |
| Ankle Posterior Tibialis PD | 0.648 | 0.19-2.25 | 0.495 |
| Ankle Peroneal GS | 0.299 | 0.035-2.58 | 0.272 |
| Ankle Peroneal PD | 0.299 | 0.035-2.58 | 0.272 |

ECU. Extensor carpi ulnaris; GS: Gray scale; PD: Power Doppler; TS: tenosynovitis.

# Supplementary Table S14. Principal component analysis of clinical and serological variables for all patients

| **Clinical and serological**  **variables** | **Component** | | |
| --- | --- | --- | --- |
|  | 1 | 2 | 3 |
| **ACPA low-positive^a^** | **0.925** |  |  |
| ACPA high-positive^b^ | 0.912 |  |  |
| RF low-positive^c^ | 0.873 |  |  |
| RF high-positive^d^ | 0.864 |  |  |
| **Swollen joint count ≥ 6** |  | **0.838** |  |
| Tender joint count ≥ 6 |  | 0.761 |  |
| Early morning stiffness duration ≥ 60 mins |  | 0.616 |  |
| **Symptom duration ≥ 6 weeks** |  |  | **0.783** |
| Age ≥ 60 years old |  |  | 0.744 |

Rotation method: Varimax with Kaiser Normalization. Factor loadings of <0.400 are suppressed to facilitate interpretation. The variable with the highest loading factor from each component is highlighted in bold. . ^a^ACPA >7 EU/ml, ^b^ACPA >21 EU/ml, ^c^RF > 20 IU/mL, ^d^ RF > 60 IU/mL.

# Supplementary Table S15. Principal component analysis of US variables for all patients

| **US variables** | **Component** | | | | | | | | |
| --- | --- | --- | --- | --- | --- | --- | --- | --- | --- |
|  | 1 | 2 | 3 | 4 | 5 | 6 | 7 | 8 | 9 |
| **MCP 2 PD** | **0.791** |  |  |  |  |  |  |  |  |
| MCP 3 PD | 0.774 |  |  |  |  |  |  |  |  |
| MCP 3 GS | 0.755 |  |  |  |  |  |  |  |  |
| MCP 1 GS | 0.746 |  |  |  |  |  |  |  |  |
| MCP 1 PD | 0.731 |  |  |  |  |  |  |  |  |
| MCP 4 GS | 0.727 |  |  |  |  |  |  |  |  |
| MCP 2 GS | 0.711 |  |  |  |  |  |  |  |  |
| MCP 4 PD | 0.702 |  |  |  |  |  |  |  |  |
| **PIP 2 GS** |  | **0.767** |  |  |  |  |  |  |  |
| PIP 3 GS |  | 0.757 |  |  |  |  |  |  |  |
| PIP 2 PD |  | 0.755 |  |  |  |  |  |  |  |
| PIP 5 GS |  | 0.742 |  |  |  |  |  |  |  |
| PIP 3 PD |  | 0.734 |  |  |  |  |  |  |  |
| PIP 5 PD |  | 0.726 |  |  |  |  |  |  |  |
| PIP 4 PD |  | 0.588 | 0.451 |  |  |  |  |  |  |
| PIP 4 GS |  | 0.527 | 0.470 |  |  |  |  |  |  |
| **PIP 1 PD** |  |  | **0.845** |  |  |  |  |  |  |
| PIP 1 GS |  |  | 0.809 |  |  |  |  |  |  |
| **Digit flexor tendon GS** |  |  |  | **0.850** |  |  |  |  |  |
| Digit flexor tendon PD |  |  |  | 0.848 |  |  |  |  |  |
| **MTP 3 PD** |  |  |  |  | **0.781** |  |  |  |  |
| MTP 2 PD |  |  |  |  | 0.750 |  |  |  |  |
| MTP 3 GS |  |  |  |  | 0.669 |  |  |  |  |
| **ECU tendon PD** |  |  |  |  |  | **0.813** |  |  |  |
| ECU tendon GS |  |  |  |  |  | 0.813 |  |  |  |
| Shoulder tendon PD |  |  |  |  |  | 0.567 |  |  |  |
| Shoulder tendon GS |  |  |  |  |  | 0.542 |  |  |  |
| **MTP 5 PD** |  |  |  |  |  |  | **0.897** |  |  |
| MTP 5 GS |  |  |  |  |  |  | 0.871 |  |  |
| **WRIST GS** |  |  |  |  |  |  |  | **0.882** |  |
| WRIST PD |  |  |  |  |  |  |  | 0.834 |  |
| **MCP 5 GS** |  |  |  |  |  |  |  |  | **0.722** |
| MCP 5 PD | .412 |  |  |  |  |  |  |  | 0.688 |

Rotation method: Varimax with Kaiser Normalization. Factor loadings of <0.400 are suppressed to facilitate interpretation. The variable with the highest loading factor from each component is highlighted in bold. ECU: Extensor carpi ulnaris; GS: Gray scale; PD: Power Doppler.

# Supplementary Table S16. Variables included in the forward step logistic regression model

| **Variables included in logistic regression model** | |
| --- | --- |
| Clinical and serological variables | US variables |
| ACPA positivity  Swollen joint count-66 ≥ 6  Symptom duration ≥ 6 weeks | MCP 2 PD  PIP 2 GS  PIP 1 PD  MTP 3 PD  MTP 5 PD  WRIST GS  MCP 5 GS  Digit flexor tendon GS  ECU tendon PD |

ECU: extensor carpi ulnaris; GS: Gray scale; PD: Power Doppler.

# Supplementary Table S17. Multi-variate regression of joint US variable with tendon US.

| Combination of variable | **Variable added** | **P*** | **Nagelkerke**  **R^2^** | **% patients correctly identified**  **(RA vs. non-RA)** |
| --- | --- | --- | --- | --- |
| ACPA positivity  DF tendon | GS positivity of MCP 1 | 0.02 | 0.464 | 78.5 |
| ACPA positivity  DF tendon | GS positivity of MCP 2 | 0.099 | 0.401 | 78.5 |
| ACPA positivity  DF tendon | GS positivity of MCP 3 | 0.022 | 0.424 | 79.4 |
| ACPA positivity  DF tendon | GS positivity of MCP 4 | 0.019 | 0.425 | 77.6 |
| ACPA positivity  DF tendon | GS positivity of MCP 5 | 0.032 | 0.417 | 77.6 |
| ACPA positivity  DF tendon | GS positivity of one or more MCP 1-5 | 0.009 | 0.451 | 78.5 |
| ACPA positivity  DF tendon | PD positivity of MCP 1 | 0.001 | 0.477 | 79.4 |
| ACPA positivity  DF tendon | PD positivity of MCP 2 | 0.095 | 0.401 | 77.6 |
| ACPA positivity  DF tendon | PD positivity of MCP 3 | 0.008 | 0.439 | 80.4 |
| ACPA positivity  DF tendon | PD positivity of MCP 4 | 0.143 | 0.395 | 76.6 |
| ACPA positivity  DF tendon | PD positivity of MCP 5 | 0.011 | 0.435 | 77.6 |
| ACPA positivity  DF tendon | PD positivity of one or more of MCP 1-5 | 0.003 | 0.463 | 78.5 |
| ACPA positivity  DF tendon | GS positivity of PIP 1 | 0.012 | 0.436 | 75.7 |
| ACPA positivity  DF tendon | GS positivity of PIP 2 | 0.160 | 0.393 | 76.6 |
| ACPA positivity  DF tendon | GS positivity of PIP 3 | 0.185 | 0.392 | 77.6 |
| ACPA positivity  DF tendon | GS positivity of PIP 4 | 0.065 | 0.407 | 76.6 |
| ACPA positivity  DF tendon | GS positivity of PIP 5 | 0.011 | 0.443 | 76.6 |
| ACPA positivity  DF tendon | GS positivity of one or more PIP 1-5 | 0.038 | 0.415 | 78.5 |
| ACPA positivity  DF tendon | PD positivity of PIP 1 | 0.022 | 0.424 | 75.7 |
| ACPA positivity  DF tendon | PD positivity of PIP 2 | 0.036 | 0.415 | 77.6 |
| ACPA positivity  DF tendon | PD positivity of PIP 3 | 0.087 | 0.402 | 78.5 |
| ACPA positivity  DF tendon | PD positivity of PIP 4 | 0.114 | 0.399 | 76.6 |
| ACPA positivity  DF tendon | PD positivity of PIP 5 | 0.014 | 0.438 | 76.6 |
| ACPA positivity  DF tendon | PD positivity of one or more PIP 1-5 | 0.031 | 0.418 | 78.5 |
| ACPA positivity  DF tendon | GS positivity of MTP 2 | 0.500 | 0.380 | 76.6 |
| ACPA positivity  DF tendon | GS positivity of MTP 3 | 0.300 | 0.386 | 76.6 |
| ACPA positivity  DF tendon | GS positivity of MTP 4 | 0.633 | 0.378 | 76.6 |
| ACPA positivity  DF tendon | GS positivity of MTP 5 | 0.013 | 0.436 | 75.7 |
| ACPA positivity  DF tendon | GS positivity of one or more MTP 2-5 | 0.031 | 0.418 | 78.5 |
| ACPA positivity  DF tendon | PD positivity of MTP 2 | 0.034 | 0.419 | 75.7 |
| ACPA positivity  DF tendon | PD positivity of MTP 3 | 0.065 | 0.411 | 75.7 |
| ACPA positivity  DF tendon | PD positivity of MTP 4 | 0.357 | 0.384 | 76.6 |
| ACPA positivity  DF tendon | PD positivity of MTP 5 | 0.037 | 0.418 | 76.6 |
| ACPA positivity  DF tendon | PD positivity of one or more MTP 2-5 | 0.072 | 0.405 | 75.7 |

*The p-value corresponds to the joint US variable in each of the logistic regression model. GS: Gray scale; PD: Power Doppler; DF: digit flexor.
